# Peer mentorship for physically injured veterans in healthcare settings during ongoing military conflict: benefits and barriers

**DOI:** 10.3389/fpubh.2026.1866434

**Published:** 2026-09-17

**Authors:** Rivi Frei-Landau, Chen Katz-Even, Yulia Muchnik-Rozanov, Noam Rechtman, Amikam Cohen, Yariv Lev-Ari, Inbar Sitbon, Yoram Ben-Yehuda, Arale Dayan, Frida Huli, Shimon Navon, Aharon Karov, Tal Sultan, Shira Ashkenazi, Yossi Gindi

**Affiliations:** 1 Department of Psychology, Ariel University, Ariel, Israel; 2 Department of Psychiatry, Schneider Children’s Medical Center, Petah Tikva, Israel; 3 Faculty of Education, Achva Academic College, Havat Shikmim, Israel; 4 Casualties Division, Israel Defense Forces, Tel Aviv-Yafo, Israel; 5 Drachim Institute for Mental health, Rehovot, Israel; 6 School of Social work, Tel Aviv University, Tel-Aviv, Israel

**Keywords:** peers with lived experiences, qualitative study, rehabilitation, self-determination theory, social learning theory, traumatic injury

## Abstract

**Background:**

Peer mentorship by individuals with lived experience is increasingly recognized as supporting recovery after severe injury. Yet evidence on its role in hospital-based rehabilitation remains limited, particularly in cases of severe war-related physical injury during ongoing military conflict. This study examines the benefits and barriers of peer mentorship within the Israel Defense Forces Casualties Affairs Unit, which trains previously injured veterans to accompany newly injured soldiers during hospitalization in healthcare settings as part of their reserve duty.

**Methods:**

The study employed a qualitative design, comprising 41 in-depth interviews: 21 with severely injured soldiers (with amputations, traumatic brain injury, spinal cord injury, burns, polytrauma) who were assigned peer mentors, and 20 with parent-caregivers. Interviews underwent linguistic and thematic analyses.

**Results:**

Peer mentorship was found to support recovery across three domains: functional (independent functioning, exercising rights, community participation), social–emotional (fostering hope, belonging, trust, and mediation of re-entering the public space), and cognitive (autonomous choice, insight, identity reconstruction, and developing an effective mindset when interacting with others). Barriers were also identified across three domains: technical-functional (matching and timing issues), emotional (managing rejection, shattering the illusion of full recovery, appearance-related avoidance), and cognitive (discrepancies in worldviews).

**Conclusion:**

Integrating peer mentorship into the post-acute continuum of care facilitates recovery of severely injured soldiers and their families but requires careful implementation. Healthcare systems and rehabilitation units may integrate peer mentorship as part of their comprehensive support framework. The study’s significance lies in the need to develop diverse responses for war casualties following military conflicts worldwide.

## 1 Introduction

The October 7th, 2023 war between Israel and Hamas, which began with a large-scale cross-border attack on communities and military posts in southern Israel and developed into a prolonged, multi-front military conflict, has resulted in multiple casualties and traumatic loss (1, 2). By April 2026, 6,424 soldiers were physically wounded, of whom 2,530 were seriously and moderately wounded (3), underscoring an urgent need for evidence-based interventions for the injured persons and their families. In recent decades, Peer mentorship by individuals with lived experience is increasingly recognized as supporting recovery after severe injury (4). Yet evidence on its role in hospital-based rehabilitation remains limited, particularly in cases of severe physical injury during ongoing military conflict. To fill this gap, the study examined the benefits and barriers of an Israel Defense Forces (IDF) peer-mentoring program for veterans with physical injuries (where previously injured veterans serve as peer rehabilitation mentors for the newly wounded as part of their reserve duty). Peer mentoring in mental and physical rehabilitation may be part of a holistic treatment.

### 1.1 Rehabilitation following physical injury

Traumatic injuries significantly impact health and mental status (5, 6). Physical trauma is a sudden potentially fatal body injury that requires urgent medical intervention. The most common types of injuries sustained in war are limb loss, spinal cord injury (SCI), traumatic brain injury (TBI), and post-traumatic stress disorder (PTSD) (7). Presence in the battlefield environment poses significant challenges (8), and an unexpected physical injury may be experienced as traumatic; thus, is associated with an increased risk of post-traumatic stress disorder, anxiety, and depression (9). A study among Israeli combat-wounded soldiers revealed that, in addition to the physical injury, they may experience the loss of self-identity and self-efficacy (10).

Rehabilitation following severe war-related injury is often prolonged and complex, underscoring the need for multidimensional rehabilitation responses (11, 12). Studies have found that social support is a major factor in post-injury recovery (13). Specifically, social support was associated with wounded soldiers’ re-integration into the community (14) and with growth, well-being, and adaptive functioning (15). Subsequently, in recent decades, social support interventions have been developed, including peer mentoring (16).

### 1.2 Peer mentorship and rehabilitation

The term “peer specialists” or “peers with knowledge from experience” refers to individuals who use knowledge derived from their own lived experience in facing adversity to support others’ recovery (17, 18). While traditional perception has viewed people with disabilities as “disadvantaged,” the psychosocial rehabilitation movement strives to create an inclusive society and recognize the power of ‘knowledge from experience’ as part of a person-centered, recovery-oriented rehabilitation approach (19, 20). Peer support involves an experience-based relationship with no hierarchical therapist-patient relationship (21, 22). Thus, beyond a therapeutic program, peer support is a movement toward independence as it positions peer mentors as productive–an aspect that has an empowering effect on their identity, and thus contributes to their own rehabilitation (23). Worldwide, peer mentoring has become increasingly common in assisting various populations, such as civilians with spinal cord injuries or acquired brain injury (24), prostitution (20), substance use (25, 26), and trauma (22).

Studies have shown that the support provided through peer-mentoring is classified into three categories: informative (counseling and sharing information and resources), emotional (providing empathy, understanding and recognition of needs), and strengthening self-worth (27). Studies among veterans have found that peer support provides social support, a sense of meaning, normalization of symptoms, and hope, while facilitating the decision to approach mental health treatment. However, challenges in creating trust, incompatible personality traits or prejudices regarding religion or race have sometimes been described (28). A recent Israeli study (23) examined a mentoring pilot program of 17 IDF wounded veterans who volunteered to accompany 25 newly wounded soldiers. The mentees reported reduced loneliness, effective integration into rehabilitation, and strengthened relationships with family members. The present study examines the benefits and barriers of peer mentoring in a larger scale program (with over 200 mentors), carried out as a reserve duty and implemented in a hospital setting within a unique context of multiple injuries. Health behaviors, including help-seeking, willingness to disclose distress, and the meanings attached to disability and to military service, are shaped by cultural context (29). The Israeli setting differs in several aspects from the volunteer-force contexts in which most peer-support research has been conducted: military service is compulsory at the age of 18 years, and the military is a central social institution, severely injured soldiers are often publicly recognized figures, and families are typically closely and continuously involved in care. Findings from peer-support programs developed elsewhere therefore cannot be assumed to transfer directly, and examination of peer mentorship within this context is warranted.

## 2 Theoretical frameworks

Two frameworks inform our interpretation of the findings. These frameworks were not used to structure data collection or coding, which were inductive. They were applied after theme development, as interpretative lenses through which the pattern of findings could be understood. Self-Determination Theory (SDT) holds that optimal functioning depends on the extent to which the social environment supports three basic psychological needs: autonomy, a sense of ownership over one’s actions; competence, a sense of mastery; and relatedness, a sense of meaningful connection to others. Where these needs are supported, motivation becomes more autonomous and internalized, which in turn promotes persistence, psychological well-being, and adaptive functioning (30). Bandura’s Social Learning Theory holds that people acquire knowledge, skills and coping strategies by observing others, particularly models perceived as similar to themselves, and identifies four sources of self-efficacy: mastery experience, vicarious experience, social persuasion, and physical and emotional states (31).

Research questions:

1. What are the benefits of peer mentoring in a hospital-based rehabilitation process following severe physical injury, as perceived by recently physically wounded soldiers and their parent caregivers?
2. What are the barriers/challenges of peer mentoring, as perceived by recently physically wounded soldiers and their parent caregivers, including both barriers to engaging with the program and challenges encountered during the mentoring process?

## 3 Method

### 3.1 Research design

The research employed a qualitative methodology, which enables examination of participants’ subjective experience (32). This methodology is suitable and widely used in the study of special populations (33) and stressful situations such as loss (34), health and rehabilitation (35, 36).

The study was conducted from a critical realist ontological position and a contextualist epistemological position. We take severe injury, its functional consequences, and the mentoring relationship to be real and consequential in participants’ lives; at the same time, we hold that participants’ accounts of them are situated, meaning-laden constructions produced within a particular cultural, institutional and interview context, and that our analysis constitutes an interpretative rendering of those accounts rather than a neutral discovery of what is “in” the data.

This position informed data collection: the interview opened with a broad, non-directive prompt so that participants could establish their own framing before the researchers’ concerns were introduced, and interviewers were trained to follow participants’ terms rather than impose program terminology.

The same position informed both analytic aspects. Reflexive thematic analysis and Systemic Functional linguistic analysis differ here in analytic gaze rather than in ontology or epistemology: the former interrogates what participants conveyed about the mentoring relationship and its consequences, the latter how their lexical choices (intensification and reiteration) encoded degrees of emotional investment. Both treat language as constitutive of, rather than transparent to, experience, and both treat the analyst as an active producer of meaning. They are therefore compatible within a single contextualist frame, and were used to build a richer account of the same phenomenon rather than to corroborate one another as independent measures.

### 3.2 Research context–the “continuing life” unit

The research was carried out in the “Continuing Life” unit, which trains wounded IDF veterans to serve as rehabilitation mentors for the recently wounded soldiers. The program was founded by Lt. Col. Yariv Lev-Ari in April 2022, and following the war was established as a military unit under the IDF’s Casualty Brigade, in collaboration with the Rehabilitation Division, Ministry of Defense. The program’s distinctive feature is that wounded veterans who, following medical discharge after their own injury, had lost their formal affiliation with the military, are re-recruited to the IDF and serve as rehabilitation mentors within the framework of reserve duty. The unit includes two tracks: (1) physical injuries, including limb amputations, head injuries, burns, spinal cord injuries, sensory impairments, and polytrauma; and (2) psychological injury–veterans with post-traumatic stress disorder (PTSD).

As studies among US veterans (28), found that they preferred that support be provided according to the injury profile, such as type of trauma (e.g., sexual trauma vs. combat trauma), gender, and even by region of service (e.g., Vietnam vs. Iraq), the unit follows the principle of matching according to injury profile. The scope of the formal mission lasts 6–8 months on a bi-weekly basis, after which the relationship may continue informally. During the formal period, the mentors participate in group meetings designed to support their own well-being as well as to discuss the challenges of mentoring the recently injured. In addition, the unit includes an extensive envelope of counselors who provide guidance in exercising rights, financial issues and accessibility. To date, eight cohorts of physical *peer mentors* (*N* = 78) and six cohorts of psychological *rehab-associates* (*N* = 84) have been trained. They accompanied 151 severely physically injured soldiers and their families, and 367 soldiers with PTSD.

In Israel, military service is compulsory for both men and women from the age of 18, and the majority of severely injured conscripts are unmarried young adults who live in, or return to, the parental home during rehabilitation. Israeli family norms, together with the practices of the IDF Casualties Affairs Unit and the Ministry of Defense Rehabilitation Division, mean that parents are typically present throughout hospitalization and are formally recognized as partners in the rehabilitation process. They function as informal caregivers alongside state-funded professional rehabilitation care, which severely injured soldiers receive irrespective of family circumstances. Where the injured person was married, the spouse fulfilled the primary caregiver role; in the present sample this applied to one participant.

### 3.3 Research population

The study included 41 participants, of whom 21 were physically injured during the October 7th War and were peer mentored through the program (soldiers with amputations, head injuries, sensory impairments, burns, polytrauma) and 20 parents/primary caregivers. Of the wounded, 19 were men and 2 were women aged 21–59. Of these, 11 were conscripts (mandatory service) and 10 were reservists. Caregivers were 15 mothers, four fathers and one spouse. The time since the injury ranged between 6 *months* to 2 years and the inclusion criterion was that mentoring period lasted at least 6 months. The decision to interview the caregiver family members alongside the injured soldiers stems from the literature that indicates that the family contributes significantly to the individual’s recovery (37). Tables 1, 2 present the demographics of the study participants.

**Table 1:** Demographic details of the physically injured study participants.

Pseudonym	Age	Gender	Injury type	Time elapsed since injury in months
Guy	23	Male	Leg amputation	14
Sasha	23	Male	Leg injury	11
Michael	28	Male	Chest injury	12
Amichai	25	Male	Polytrauma + blindness	10
Gadi	56	Male	Leg amputation	6
Rotem	24	Male	Polytrauma	6
Roy	23	Male	Polytrauma+ double Amputation	14
Bashir	21	Male	Polytrauma	15
Moshe	23	Male	Head injury	26
Binyamin	23	Male	Amputation	13
Idan	22	Male	Amputation	11
Yehuda	31	Male	Amputation	12
Yaniv	59	Male	Amputation	11
Daniel	21	Male	Burns	6
Eyal	21	Male	Head injury	11
Tal	21	Female	Polytrauma	13
Elia	24	Male	Head injury	11
Yulia	22	Female	Amputation	12
Elrom	24	Male	Polytrauma	7
Naftali	30	Male	Amputation	6
Eitan	22	Male	Polytrauma + burns	8

**Table 2:** Demographic details of the caregiver study participants.

Pseudo-nym	Age	Gender	Relationship with the injured	Injury type	Time elapsed since injury in months
Reut	63	Female	Mother	Amputation	13
Zvi	69	Male	Father	Head injury	8
Hagit	54	Female	Mother	Burns	6
Ariella	55	Female	Mother	Head injury	12
Yafit	66	Female	Mother	Amputation	13
Yair	57	Male	Father	Head injury	14
Sarit	52	Female	Mother	Head injury	12
Dor	60	Male	Father	Polytrauma	16
Simcha	53	Female	Mother	Polytrauma	14
Mika	53	Female	Mother	Head injury	27
Ronit	52	Female	Mother	Amputation	15
Rachel	46	Female	Mother	Leg amputation	16
Keren	50	Female	Mother	Head injury	6
Nurit	50	Female	Mother	Head injury	8
Vered	53	Female	Mother	Head injury	6
Moshe	60	Male	Father	Polytrauma	10
Michal	53	Female	Wife	Leg amputation	6
Dana	55	Female	Mother	Polytrauma + head injury	8
Dalia	49	Female	Mother	Head injury	11

#### 3.3.1 Rationale for a multi-perspective design

The study employed a multi-perspective qualitative design, interviewing both injured soldiers and their primary caregivers. This was not intended to yield proxy reports: caregivers were not asked to speak on the injured person’s behalf. Rather, the intervention under study is explicitly directed at the family as well as at the injured individual: mentors are trained to provide information to caregivers, to mediate parent–child communication, and to support the family through the acute hospitalization period, so that an account based on mentee data alone would omit a substantial part of what the program does. Caregivers were also present for periods of hospitalization that mentees, particularly those with TBI, could not themselves recall. The two datasets were coded separately and compared. In the findings, each extract is attributed to its source, whether an injured participant or a caregiver. In practice, contradictions between the accounts of injured participants and those of their caregivers were rare. The two perspectives proved predominantly complementary, each illuminating a different facet of the same phenomenon: injured participants described the mentoring relationship from within, whereas caregivers observed it, and its effects on the family, from an adjacent vantage point.

### 3.4 Procedure and ethics

The study was approved by the Ethics Committee of the academic institution (No. 0224; masked for blinded review) and was conducted in coordination with the authorized bodies in the IDF. All participants signed a written informed consent. The study used purposeful sampling, so it was offered only to the wounded accompanied by the unit. To avoid pressure to participate, the interviews were conducted by interviewers who are not members of the Unit. For ethical reasons, emphasis was placed on the interviewers’ tailored training, so that they could respond appropriately in case of distress. Furthermore, the parents of the wounded were interviewed by an M.A. rehabilitation psychology student with a background as a casualty officer and the soldiers were interviewed by an M.A. rehabilitation psychology student who himself served in the army. In addition, it was clarified that participation is voluntary and that they could terminate the interview at will without consequences. At the end of each interview, the interviewer checked how the interviewee felt and offered the option to contact the research team later if necessary. In practice, all interviewees felt good at the end of the interviews, and most even thanked the interviewer for the opportunity to make their voices heard. The interview location was chosen by the interviewees (face-to-face, Zoom platform or by phone). The interviews were transcribed and kept confidential. The raw material was analyzed by the researcher and the research team and was not transferred to any other party. To protect the privacy of the participants, in the findings section details that may violate confidentiality were disguised, and pseudonyms were used (for the injured person, the parent, and the mentor). In addition, identifying details that could reveal participants’ identities were disguised, given that some of the injury incidents had been reported in the media.

### 3.5 Data collection

Semi-structured in-depth interviews were conducted. The two-stage interview began with an open-ended question (“Tell me about your experience with X (the peer-mentor)”), after which the interviewees were asked additional questions. This procedure allows the interviewees to first share what is meaningful to them and then delve into other issues (38–40). The interviews lasted 30–90 min, depending on the interviewee’s request. After conducting 41 interviews, the saturation criterion was reached (41).

### 3.6 Data analysis

Multilevel analysis integrated thematic analysis with linguistic analysis. Combining methods allowed each strand to illuminate a different aspect of the same accounts (42). During the *reflexive thematic analysis* (43) the research team developed themes through sustained engagement with discourse segments that reflect the benefits and barriers of peer mentorship. The data were then read to identify meaningful units using inductive coding. Throughout the process, the data were read repeatedly in order to deepen and refine the developing themes and their conceptualization. This involved three separate analysts (the study supervisor – Author 1, an M.A. student, and a research assistant), following which joint brainstorming was conducted.

The analysis proceeded through the six phases of reflexive thematic analysis. (1) Familiarization. All three analysts read the full transcript set before coding, listening to recordings where available, and recorded initial impressions in analytic memos. (2) Coding. Coding was inductive, complete rather than selective. Codes generated from the functional data included, for example, “asking a question without embarrassment”, “he knows what actually works,” ‘equipment brought and tried out’, and ‘advice accepted from him but not from me’ in the caregiver data. (3) Generating initial themes. Related codes were clustered into themes; the four codes above were clustered into the candidate theme “the credibility of experiential knowledge.” (4) Developing and reviewing themes. Candidate themes were checked against the coded extracts and the full dataset. “The credibility of experiential knowledge” was found to cut across functional, social–emotional and cognitive material rather than to constitute a domain-specific theme; it was therefore retained as an organizing insight in the Discussion, while its functional content was developed into the theme “Promoting independent physical functioning.” Conversely, an early candidate theme, “bureaucracy,” proved too thin and descriptive and was developed into “Financial conduct and exercising rights” by incorporating latent material about the relief of delegated administrative burden. (5) Refining, defining and naming themes. Each theme was given a definition and a boundary statement specifying what it did and did not include. (6) Writing up. Analytic writing was treated as part of analysis, and theme definitions were further revised during drafting. Consistent with reflexive thematic analysis, the discussions between analysts were used to deepen and complicate interpretation rather than to establish coder agreement; we therefore do not report an inter-rater reliability statistic.

*Linguistic analysis* was employed to explore language choices throughout the interview data (44), allowing for identification of implicitly communicated messages while content analysis supported the organization and interpretation of participants’ explicitly expressed views and perceptions (45). The linguistic analysis was informed by Systemic Functional Linguistics (46), offering a framework for examining how specific lexical choices tend to create emotionally-colored language and convey emotional meaning (ibid). In particular, the use of intensifiers when describing emotionally significant experiences can reflect varying degrees of emotional investment. These intensifiers may be “interpersonally neutral,” such as *very*, *really*, *completely*, or they may convey evaluative judgments, for instance, *amazingly* or *unbelievably* (ibid., p. 356). The presence of such intensifiers often points to experiences or ideas that the speaker viewed as especially meaningful (46). Repetitions are also seen as a form of intensification, emphasizing particular ideas or experiences. Based on this approach, in the caregiver’ interviews, we identified utterances marked by intensification—whether through adverbial intensifiers or repetition—as a means of expressing the caregivers’ emotions as well as the marker of more emotionally intense experiences than those described without the intensifiers. Descriptions that included the adverbs *very*, *terribly*, *many*, *much*, *indeed*, *absolutely*, *definitely*, *totally*, etc., were coded as instances of linguistic intensification. The instances of lexical reiterations were coded when the same lexemes or groups of lexemes were repeated to emphasize the quality of experience or the importance of a certain idea. The analysis was conducted in two phases. First, AntConc software was used to identify relevant linguistic markers. These excerpts were then imported into ATLAS, in which we employed qualitative content analysis to identify utterances in which caregivers portrayed the peer-mentorship program and subsequently interpreted the experiences and ideas they emphasized. This phase was independently conducted by the first and third authors: the first author holds a Ph.D. in Psychology, and the third author holds a Ph.D. in Linguistics with extensive qualitative linguistic analysis experience. To ensure credibility and trustworthiness, each researcher analyzed the data separately and then collaborated in reflective discussions. Differences in interpretation were explored in discussion and used to develop a richer reading of the data. Table 3 summarizes the coding method of linguistic analysis.

**Table 3:** Linguistic analysis and coding method.

Distinctive linguistic marker	Subcategories	Coding method	Examples
Intensification	Adverbial	Adverbs indicating higher or lower intensity by emphasizing the quality of the experience	It can really be a good thing of that kind of parent mentor
	Lexical repetition	Repeating one or more lexemes to emphasize the quality of the experience	I have no complaint, criticism or any kind of question about his functioning; he put his heart and soul into it. Really, his heart and soul.

### 3.7 Trustworthiness

Multiple analysts engaged with the data in coding, analyzing, and interpreting it, in order to broaden and complicate interpretation rather than to converge on a single reading (47). Moreover, ‘prolonged engagement’ was created through lengthy interviews, which supported the production of detailed and candid accounts. In addition, consultation was carried out with colleagues who have lived experience of injury (expert-by-experience consultation). Finally, the principle of reflexivity was applied. Accordingly, the facilitator and interviewers constantly examined their preconceptions, values, and how their interpretations were influenced by these aspects, in order to remain accountable to participants’ accounts and to make their own influence on the analysis visible.

### 3.8 Reflexivity

The research team occupied a partly insider position. The first author serves as a reserve psychologist within the “Continuing Life” program and was involved in the mentors’ supervision groups; the third, fourth and fifth authors had no affiliation with the unit. This positioning afforded access, contextual understanding and participants’ trust, but also carried an evident risk of favorable bias toward the program. Several steps were taken in response. Interviews were conducted by two M.A. rehabilitation psychology students external to the unit (fourth and fifth authors), who were briefed to invite criticism explicitly and not to defend the program if it was criticized. During analysis, the authors without unit affiliation led the search for disconfirming and negative cases; the barriers reported under RQ2, including the accounts of unresolved mentor-mentee mismatch, emerged largely from that process. The first author kept a reflexive journal recording moments of discomfort with critical material, which were brought to team discussion. Positioning also shaped the interviews themselves. Both interviewers had prior military experience, one having previously served as a casualty officer, which we judged to have eased rapport around military identity and loss and to have prepared them to respond appropriately where participants became distressed. Neither held a current military role, and neither had any affiliation with the unit, so participants were not speaking to serving representatives of the system whose support they were being asked to describe.

## 4 Findings

### 4.1 The benefits of peer mentoring to the rehabilitation of physically injured combatants (RQ1)

The analysis revealed that the benefits of peer mentoring are expressed across three domains: functional, social–emotional, and cognitive, divided into self-orientation and environmental orientation. In each domain, four themes arose, for a total of 16 themes detailed below. Figure 1 displays the study’s themes, followed by illustrative quotes (all names are pseudonyms).

**Figure 1:**
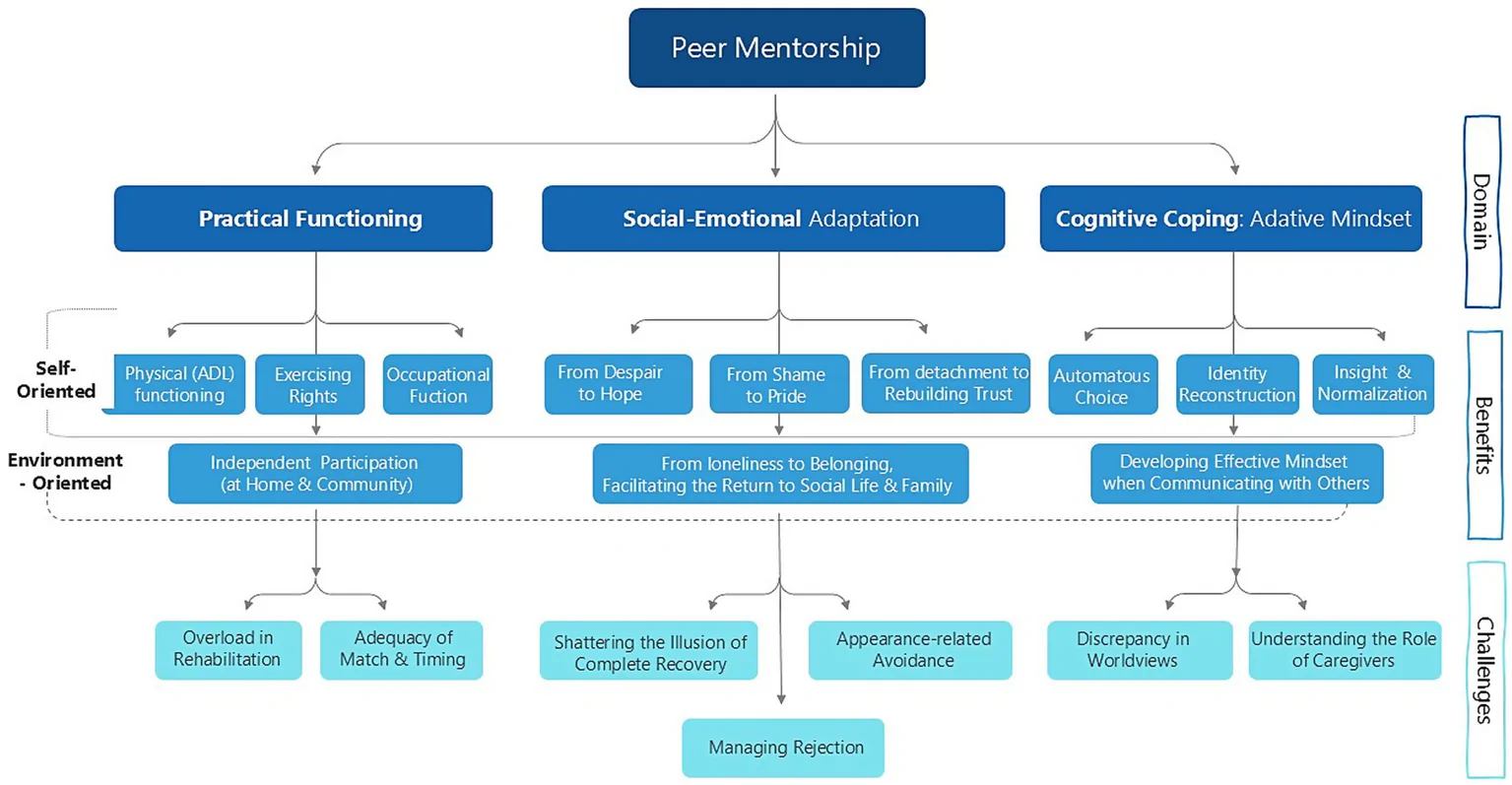
Themes denoting the benefits and barriers of peer mentorship for physically injured veterans.

We use the following definitions. The functional domain concerns capacities for practical action: performing activities of daily living, managing administrative and financial tasks, and participating in occupational, leisure and community roles. The social–emotional domain concerns the affective and relational dimensions of adjustment: hope and despair, shame and pride, trust, belonging and loneliness and re-engagement with the social world. The cognitive domain concerns appraisals, beliefs and meaning-making: locus of control, insight into symptoms, self-identity, and the mental models that guide interaction with others.

Within each domain, self-oriented themes concern changes located primarily within the injured person, in their own body, feelings, beliefs or sense of self. Environment-oriented themes concern the interface between the injured person and the surrounding world: the home, the community, public space, professionals, institutions and the family.

#### 4.1.1 Practical functioning

##### 4.1.1.1 Self-oriented: promoting independent physical functioning

Participants repeatedly described how severe physical injury challenges basic activities, which must be relearned. First and foremost, the peer mentor’s guidance provides available knowledge for practical assistance regarding issues of daily functions:

- You learn everything from scratch; walking, eating, even breathing. He (mentor) also went through the difficulties I went through. So, if I have a question–I can ask him straight away. (Elia, TBI).
- It’s like working from the ground up for everything, the basic actions that I never thought about. Often things you are told in occupational therapy-it’s advice that comes from a place of academic learning, but when someone comes with experience, he really knows what works for him in life, so it works better. (Idan, amputation).

Hospitalization is described as an overwhelming experience, involving visits by many well-intentioned people who cannot help, unlike the peer mentor, who brings knowledge from experience that is perceived as ‘golden’ knowledge:

A lot of people came. It was moving but sometimes a bit too much. But it wasn’t until I got to know Yagel [mentor] that I realized how much I needed him, and what golden knowledge he had… And he brought me piles of equipment: take it, try this. He helped me deal with the confusing world of prosthetics. My injured friends are in the same boat, they do not know any more than I do. And family is supportive, but they also do not know. If Yagel had not had an amputation, maybe he would not have helped me as much. He tells you things from experience. That’s the greatness of this program. (Yehuda, above-knee amputation).

Yehuda sees the knowledge of a mentor with a similar injury as unique, as opposed to the knowledge that can be obtained from other recently wounded soldiers or family members. Moreover, learning from the mentor how to function is not limited to verbal advice, but also by observing the mentor’s behavior. This is how Gadi describes it: *“I see how Aner* [mentor] *manages with a prosthesis – it gives me a lot!”*

Interestingly, the parents noted that when the advice came from an experienced person, it was more easily accepted by their injured child compared to the parent’s advice: *“Reuven* [mentor] *tells him: Put on these elastic bandages because otherwise you will have scars. If I say so, then I’m just a nagging mother. He can tell him things I cannot.”* (Hagit, mother).

The uniqueness of knowledge from experience not only helps the mentees, but also their caregivers. This is how Racheli, a mother of a soldier with amputations, describes it:

I was looking for bright spots… It was my life’s mission at that moment–to understand how I was helping him [injured son] to reach a full and good life. But I did not know how in practice… So, it was a ray of light in a dark time. He helped me put together a list of tasks to be done. it was very important that the information on how to do things on the practical side was made accessible.

Interestingly, Rachel’s need for bright spots’ translates into practical aspects. This aspect was repeated throughout the interviews: at the initial stage, it is the practical advice about the functional aspects that strengthens the sense of being able to cope. In addition to practical advice, the mentor provides knowledge about the injury that promotes caregivers’ insight regarding the consequences, especially when these are “transparen”, as in a head injury:

Nir [mentor] explained us what it means to have a head injury practically… One day we had to move to a different room, and the closet had to be tidied up. So, he said to me: ‘Do not touch. Let him do it, I’ll guide him if necessary. He guided him on how to tidy up. (Nurit).

##### 4.1.1.2 Self-oriented: financial conduct and exercising rights

Alongside the peer mentor’s contribution, parents also mentioned the contribution of the counseling team that assisted in exercising rights: *As a mother of someone with a head injury you do not understand what your rights are, and how to deal with bureaucracy. And they come and help*. (Ariella, mother, TBI). In the face of the overwhelming emotions and the anxiety about the injured son’s health, the counselors are described as helping the parent reorganize:

When your son is fighting for his life–it’s Too Much Information!. I felt that they are organizing the chaos for me… Everyone comes wholehearted, but they come with the goal of putting things in order for you. (Vered, mother).

Ultimately, assistance in exercising rights not only helps the parent but also allows the injured soldier to focus solely on their rehabilitation process:

After a month of rehabilitation, I really fell apart. I fell apart completely… The hardest thing is that you gave your life to the country. True, I chose to give, but you expect the state to help you. But what turns out is that they tell you about 50 percent of the rights, another 25 percent you know from the other guys in rehab, and another 25 you have no idea… And then he came and said to me: “This and that are your rights.” He opened a WhatsApp group for me and that’s where all my problems were solved… That’s what I needed. Without it I could not function, I collapsed. (Elrom, polytrauma).

I know it really changed my life. It gave me the freedom to deal with the rehabilitation from my injury. Because they took care of that aspect. (Moshe, TBI).

Some of the wounded believed that the support they received was a factor that ultimately expedited and shortened their rehabilitation period: *I did not know what my rights were, my business collapsed. They helped me a lot. Also, on the emotional side… I’m sure that my time in rehab has been shortened thanks to this help. (Elia, TBI).*

##### 4.1.1.3 Self-oriented: promoting occupational and leisure functioning

Many participants referred to the connection created through the mentor to additional frameworks–whether leisure or therapeutic–such as attending Beit HaLochem [major community rehabilitation center], going out together for sports activities. For example, Guy, with a leg amputation, noted: “My mentor, I connected with him a lot. He takes us kayaking. It really helps.” Some of them also mentioned assistance with employment or applying for university. Noteworthy, that this aspect emerged only at a later stage, after the return to the community; thus, was less frequent.

Moreover, the mentor and the program’s counselors helped connect the family to official assistance agencies in the community, through which the injured person could receive support in integrating into leisure and occupational activities. For example, Nurit, mother of a soldier with a head injury, describes how the program’s counselors approached the local council where the family resides in order to enlist its help: *“The municipal authority ‘woke up’ only in the last month. And that’s thanks to Amiad. He told them, ‘There’s a case here. Where are you?! Where do you fit in, and that’s helpful.*”

##### 4.1.1.4 Environment oriented: promoting independent participation at home / in the community

A key aspect described by participants as challenging relates to independent functioning upon returning home. Moshe, father of a soldier with blindness and amputations, says: *I want Amichai [injured] to have an option to come home, but his childhood room is irrelevant.* In this context, accessibility based on personal experience combined with professional knowledge creates efficiency. This is how Racheli, whose son lost a limb, describes it:

I made it my goal to get home for the holiday, and that was my mission–how do I make the house accessible… I did not care what anyone thought of me. I knew they thought I was unrealistic. So, they came to my house and helped start an accessible home project.

Relatedly, Gadi, who lost a limb, described how the consultants helped him obtain handicapped parking near his home: *“And they helped with applying for disabled parking near the house. We do not always know who to turn to and how.”* Others received assistance in applying for a driver’s license or making an informed choice of the prosthesis selection, all of which promote independent functioning in the community. Yafit, whose son lost a leg, describes it: *He also helped him with administration, in obtaining a driver’s license after his injury. He even came to the institute in San Diego when he was given a different prosthesis.* Interestingly, practical assistance not only contributes to functioning but it also facilitates a sense of trust, creating connection, and instills confidence. Amichai, who has blindness and amputations, says:

They helped me with practical things. It’s like an anchor. Let me tell you something, most of my questions are relate to something practical: Bureaucracy, exercising rights, things like that. But, out of all the practical help, eventually it’s a lot of emotional stuff because it gives me so much confidence-knowing that I have people I trust, …who understand from experience. It gives me a lot of confidence. A lot!

It seems that assistance with practical issues constitutes an interface between the functional and emotional levels, as described below.

#### 4.1.2 Social–emotional adaptation

##### 4.1.2.1 Self-oriented: from despair and helplessness to hope

Encountering a veteran with a similar injury who has adapted and lives a full life provides the recently injured and their parents with hope, inspiration and confidence in their ability to cope:

It gave me faith that if he could do it, so could I. Because he has the same injury as mine. He also told me: It’s a serious injury, but it can be overcome. He gave me an example of himself and the marathons he ran. (Eyal, TBI).

It helped me tremendously, especially at the beginning…Because you know what injury looks like now, but you do not know what life will look like after that. And to see someone who has undergone something very similar but already has perspective. He worked full-time and was a Paralympic athlete and he is a father… everything that an ordinary person wants and experiences in life, plus. It gave me a lot of inspiration. (Idan, amputation).

The program mainly gets the family past the initial shock. They help them understand that there is life after the injury. (Sasha, polytrauma).

These participants stressed the importance of timing, especially during the initial shock, when uncertainty and helplessness are most acute. In this context, meeting a mentor serves as a metaphorical encounter with an optimistic image of the future, thereby enhancing hope:

You see him, and you see the future. You see that one can overcome this, that it’s possible to be rehabilitated. Even though at first it seemed like it would never be possible…You feel it’s possible to get through it.

Injury-profile similarity appears to facilitate identification with the mentor, who is perceived as a role model, thereby enhancing hope and self-efficacy. Moreover, meeting the mentor fosters hope not only for the injured individual, but also for parents and spouses:

I think it really helped my parents understand that my life would be more complicated, but I would have a life. (Bashir).

Shmulik [mentor] introduced me to his wife… He invited me to come to their home with Oshrat, my girlfriend, who would also see that it is possible to live a good life like this. (Amichai).

As noted, fostering hope among family members indirectly benefits the injured person, as relationships are central to quality of life.

##### 4.1.2.2 Self-oriented: from shame and guilt to pride and recognition

Injury may evoke shame or guilt regarding its circumstances, which may hinder rehabilitation:

It’s hard for me with the friends who are gone and I’m here. They were more righteous than me… I spoke to him [mentor] freely about everything. I learned from him not to be ashamed, to be proud. Do not hide. I made sacrifices and I have no reason to be ashamed of my disability. It helps to talk to someone you identify with. (Daniel).

Daniel described survivor’s guilt and shame related to his disability, but sharing these feelings with his mentor helped foster pride and self-recognition.

##### 4.1.2.3 Self-oriented: from detachment to rebuilding trust and continuity

Participants described the difficulty of being forced out of the military system following injury. For example, Yaniv, a reservist who underwent amputation, remarked: “*If you are not healthy, they do not need you. That’s how the army is*.” At the same time, the mentor’s military affiliation and deployment by the IDF helped restore trust in the system. Some participants also viewed the program’s military framework positively, perceiving the support as free of ulterior motives, unlike some non-profit organizations (NPOs). A number of NPOs in Israel provide services, delegations and recreational programs to wounded veterans; some also involve wounded individuals in public and fundraising activity, which several participants experienced as instrumental: *It’s good that this is through the army and not an NPO. It gives the feeling that it is pure. He does not profit at my expense and does not take advantage of me*.

Another factor that fosters trust is the mentor’s sustained presence over time, particularly through transitions between care settings and the return to community life:

The problem with injury is that you do not stay in the same institution or department, you move between so many places. I think we moved between seven departments. What’s special about this program is that Lior [mentor] accompanied us no matter where we were… that’s the only way to create a relationship that you can trust. (Ariella, mother, TBI).

Interestingly, many participants noted that informal contact continued even after the formal mentoring period had ended: “*We connected and till day we are friends. We would sit and talk”* (Bashir, polytrauma).

##### 4.1.2.4 Environment-oriented: from loneliness to belonging, facilitating the return to social life

Discharge from hospital is gradual, beginning with day hospitalization and then transition to the community. At both transition points, the mentor helps reduce feelings of loneliness and supports renewed engagement with the social world. This is how Tali, with a gunshot wound, describes the transition from hospitalization to ambulatory treatment:

When I moved to day rehab, the loneliness was hard. In the hospital, I used to be surrounded by people all day… Efrat [mentor] showed up and we would get together for a coffee.

For some injured participants, entering public social spaces was experienced as embarrassing due to the visible consequences of injury. The mentor was described as helping them “re-enter the world” by modeling adaptive behavior in public settings. Idan, for example, described going to a restaurant with his mentor, Asher:

Encounters with the outside world are difficult. You constantly feel eyes on you. And seeing him [mentor] walking like that, fully confident, in public places… it gave me a lot *at first*. He gave me an understanding that it depends where I’m going to take it. If I look miserable, there will be pity. And if I’m confident and proud, that’s what people will see. I think that’s one of the things where at the beginning, Asher’s impact was the greatest.

By using the phrase “at first” Idan highlights the difficulty of initially re-entering the social environment as a person with an amputation. The mentor supports this process in three ways: by accompanying him in his first public outing, modeling confident behavior in public, and conveying an adaptive mindset centered on choice and self-presentation. This cognitive shift is further elaborated in the following section.

#### 4.1.3 Cognitive: adaptive mindset

##### 4.1.3.1 Self-oriented: establishing an attitude of autonomous choice

The mentor promotes insight that the injured person has autonomous choice, thereby strengthening internal locus of control and supporting more adaptive adjustment to the injury:

I feel like the biggest thing I’ve learned is the choice; the choice of how to live. Eventually, every day has the potential to be bad, if I choose it… I can choose.

Parents likewise emphasized the mentor’s central role in fostering a worldview of choice, one that aligned with their own views:

When Amichai woke up, we talked about how he could choose. He can say: “I’ve been screwed” and then demand. And he can say: “I’m miraculously alive, and it did not hurt my ability to think or my sense of humor”… A person must decide for himself. Shmulik [mentor] is such an optimistic person, and I have seen that many of his friends are like that. They stand tall in how they feel about the world. It’s a gift that they connect. It’s a gift. The social climate he finds himself in is important.

Sometimes the mentor connects the injured person to their own group, which includes other people who have coped effectively–thereby creating a social–emotional climate that supports autonomous choice. Similarly, mentoring helps promote the self-identity of a “person,” and not of a “wounded person” vis-à-vis NPOs that sometimes cynically use an injured person for fundraising, thereby establishing him as ‘wounded’ above all else:

I returned from a delegation of X (an NPO)… And he helped me with this transition back to routine. He helped me get back to being a “human being” and not being the “wounded one.” (Guy, amputation).

##### 4.1.3.2 Self-oriented: addressing the identity struggle

The injury may challenge the soldier’s self-identity, which until now was defined as a “fighter on the battlefield.” Mentors helped them reconstruct their identity from a fighter on the battlefield to fighter in rehabilitation, an identity that is no less important than his previous one.

The fighting for me ended abruptly. It’s not that I wanted to stop being a combatant, and I see friends around me continuing to fight, ah. it’s hard. So, I talked to him [the mentor] who went through this many years ago, and suddenly understood that it’s okay, I’m in a personal war, and they are in theirs… It helps to put things into proportion. (Rotem, polytrauma).

Similarly, Naftali, who has leg amputation, describes the “fighting” mentality that stemmed from the personal example of his mentor: *He gave me a personal example. I was a combat soldier in the army. And now it’s about fighting yourself. Go to treatments and rehabilitate yourself*.

##### 4.1.3.3 Self-oriented: cultivating insight into the symptoms and normalization

Some of the physically injured also experience symptoms of PTSD, which undermine their self-perception. The mentor provides a clear explanation that promotes insight, reduces anxiety and gives a sense of normalization.

Avi [mentor] copes with an amputation and has PTSD, and then he explained me what was happening to me, why I was screaming, and I realized what I was going through. I used to be the most patient, calmest person, I never yelled or fought with my family. We also talked about where you take things. What do you do with it. (Elrom, polytrauma and PTSD).

As seen, in addition to providing information about symptoms and normalizing, the mentor encourages to look for a way to effectively deal with the situation. Ultimately, this conduct strengthens the belief in his ability to cope with the situation:

Ephraim [mentor] helped me a lot with knowledge about PTSD. I ask him: ‘Is this normal?’ It’s easy to get sucked into the place of *poor me*, I do not want that. I ask him about triggers, because I’m new to this. He helps me. He directs me to sports. It gives me a horizon, and I see that it’s possible to succeed. (Michael, chest injury and PTSD).

##### 4.1.3.4 Environment-oriented: developing effective mindset when interacting with others

The mentor fosters thinking patterns that promote effective interaction with the environment. The environment includes professionals (such as a prosthetics institute), various NPOs, aid agencies as well as family. For example, Yehuda described helpful instructions about communicating with the prosthetics technician:

He helped me so much! How to communicate with the prosthetics technician, how to tell him what I need. And what should I expect now? Because prosthetic legs are a real project. (Yehuda, amputation).

Others spoke of assistance in dealing productively with offers from various NPOs that did not match their needs (e.g., joining a delegation to raise funds during a critical period of rehabilitation), as well as assistance in management in meetings with the Ministry of Defense. Another domain was the relationship with the family. The injury often induces a regression in the parent–child dynamic: the wounded son, who had left home and functioned as an independent combat soldier, suddenly becomes dependent on family members:

Our soldiers are in a place where they no longer want to be dependent on their parents, so the dialogue with them is less accessible than with a man who has gone through the same injury… Uriel [mentor] can get him to talk; he understands his needs and is open with him. It’s a huge help. (Mika, mother, TBI).

As seen, the mentor, as a buddy-to-buddy is able to conduct meaningful conversations. Furthermore, some parents received the mentor’s guidance in navigating the complex communication with their own son:

Gently, Reuven [mentor] taught me what to do. Because Eitan [injured son] does not tell me everything, Reuven made sure to keep me in the loop. He noticed my distress. He was a kind of bridge. Eitan felt that Reuven was not us and allowed himself to be helped and still feel independent. It’s not his parents, it’s his friend. And Reuven, he could have left me out of the picture, but he was gentle–he was there for both of us. (Mother, burns).

The mentor appears to facilitate interactions with the injured person’s family, particularly the parents, by providing information, mediating communication, and at times buffering conflict.

### 4.2 Barriers of peer mentoring during the rehabilitation process (RQ2)

The analysis revealed that the barriers of peer-mentoring were also expressed across three domains: practical, emotional, and cognitive. These included 7 themes, as detailed below.

The challenges and barriers reported below encompass both barriers to engaging with the mentoring program and challenges that arose during the mentoring process itself.

#### 4.2.1 Practical challenges

##### 4.2.1.1 Overload in rehabilitation

Many participants described how the intensity of rehabilitation creates an information overload and makes it difficult to understand the program’s potential contribution (as the mentorship begins during hospitalization). This is how a soldier with polytrauma describes it:

The average person in rehab is overwhelmed. People come into his room all the time and offer everything, so it’s very easy to miss this program… I was very close to saying no to the program. And what would I have lost! (with emphasis).

Another consequence of this overload was difficulty coordinating the multiple rehabilitation treatments. This may create scheduling incompatibilities that hindered relationship formation:

It’s all goodwill, but there’s something to fix… Rami [mentor] accompanies me. But he can mostly come in the morning when I’m in therapies. So, I kind of took a break.

At times, a vicious cycle emerged: difficulties coordinating meetings amid the demands of rehabilitation were interpreted by mentors as personal rejection, which could undermine persistence and weaken the relationship, as discussed below.

##### 4.2.1.2 Adequacy of match and timing

What constitutes an optimal match? Analysis identified several relevant parameters. First, similarity in injury type, as previously demonstrated in participants quotes. Second, gender matching was also found significant. For example, Yulia, who had lost a limb, described her need for a female mentor:

There are struggles that a man will not understand. For example, high heels; a prosthesis for a woman. Men do not care what kind of foot they get. I wanted an adjustable foot so that a platform could be inserted… When visitors come, they automatically skip me. They do not think I’m a soldier since I’m a woman. This is annoying. So, Idit [mentor] understood me.

Third, age matching was also found to be relevant, although personality could compensate when needed:

One of the things that helped Yagel and I connect was the fact that I was 31 years old, I was injured while doing reserve duty…We were a precise match.

It is not common that a 25-year-old man connects with Shmulik [a 60-year-old mentor]. He′s such an optimistic person. It’s a gift that they connected. (Avidan, father).

Evidently, given an age gap, personality maturity and interpersonal competence overrides the other considerations. Another practical aspect was the timing, as elaborated below.

#### 4.2.2 Emotional challenges

##### 4.2.2.1 Shattering the illusion of “complete recovery”

As noted, the mentor embodies the injured person’s possible future self. In cases of amputation, seeing a veteran with a similar injury walking independently aligns with the injured person’s and the family’s image of full recovery. A mother described it as follows: *The first time he [mentor] came in, we were shocked that he had lost a limb. He walks **normally**! That, in my opinion, helped connect. To see someone in a similar situation, and he lives a **normal** life*.

By contrast, in cases of spinal cord injury, families encounter a person using a wheelchair while still in the early stage of hoping for full recovery. This may conflict with the expectation of complete recovery and, in turn, generate resistance to engaging with the injured mentor. Indeed, difficulties in establishing connection were observed both in SCI and sometimes with TBI, depending on its severity. After conducting a focus group brainstorming with five mentors with SCI, who discussed the ideal moment to begin the mentoring, it was agreed that in the case of a SCI the placement should be performed gradually, once the injured had internalized the injury.

##### 4.2.2.2 Appearance-related avoidance

An encounter with the mentor involves an encounter with his external appearance, which is sometimes challenging. The mother of a seriously injured person describes it thus:

You know, his physical appearance. At first, I was a little shocked. We went through a process with it. We are worried our son should not look like that [crying]… On the one hand, you want him to be like him, and on the other hand, you really do not want him to look like him. So, seeing him [mentor] is both encouraging and frightening.

This quote reflects profound ambivalence: the mentor simultaneously embodied hope for recovery and a feared image of the injured son’s possible future, making the encounter both reassuring and distressing. This difficulty regarding the mentor’s external appearance was most evident in cases of severe injuries, such as burns, head injuries, and spinal cord injuries, and posed challenges to initial contact. However, the mentor’s emotional maturity often compensated for this difficulty, enabling the development of a connection.

##### 4.2.2.3 Managing rejection and reaching out

Another challenge revolved around the need to “reach out” to the injured, an aspect that was experienced as emotionally complex for some mentors. However, those who persisted were able to create a meaningful connection:

At the beginning, Regev [injured son] did not want to hear about the program and I said to Pini [mentor]: There’s a problem, he does not want to sit with you at all… I remember Pini saying: ‘do not worry, we’ll get along, I was like him too’. He came to the physiotherapy … and slowly he managed to peel off the layers and create a good relationship with him. (Ariella, mother, TBI).

In the supervision support group for mentors-on-duty, the emotional difficulty of coping with rejection was repeatedly discussed. When mentors viewed this as a personal rejection they experienced great distress, but when they were able to see how this rejection stems from a loss of trust in the world caused by the injury and/or from technical rehabilitation overload; thus, not personal, they managed to reach a close connection with the injured person.

#### 4.2.3 Cognition-related challenges

##### 4.2.3.1 Discrepancy in mentor-mentee worldviews

Potential perceptual gaps between the injured person and the mentor sometimes posed challenges. For example, socio-demographic differences occasionally raised concerns; nevertheless, through persistence and interpersonal connection, a meaningful relationship was established. As Yehuda described:

I love Yagel [mentor], he is pure gold, a good soul. And I remember that. I define myself as secular, and when he arrived, I saw that he was religious. I questioned the match. Maybe our beliefs are different? Will we connect? And we have a great connection; I simply admire him.

In other cases, the pair did not overcome the gaps, but the injured person was able to find compensation in the contact with other program consultants. Thus, for example, Roy describes:

With Sami (mentor) we are quite different. We’re less in touch and I’m fine with it. We are different. Today, the people who help me are mainly Yariv, Yossi and Tal. I meet Yossi twice a week and we became real friends. (Roy, polytrauma).

Sometimes a positive and warm relationship is created but over time there are gaps in worldviews, but these can be bridged. Thus, for example, Idan says:

I think that Asher and I are similar, but not alike. We have common interests…And on the other hand, I’m slightly more introverted, and Asher is more extroverted. There were also cases that it took me a moment to understand that there are things that are his, not mine. For example, he was trying to push me to leave the house quickly, aha. To live independently, alone. Because it suited him and how he experienced his injury. But my support system is completely different from the one he had, and I felt I needed the support of the family. there was some gap between us. And I said, we have similar struggles but it’s not like we have become the same. Still, he is like a friend to me, and I still need his advice here and there.

Idan identified a gap between himself and the mentor regarding leaving the house. However, he viewed it as a personality difference rather than incompatibility, and chose the path that suited him. Although challenging, this gap did not undermine the relationship; he later noted that he still meets the mentor and regards him as a friend.

##### 4.2.3.2 Discrepancies in perceptions of the role of caregivers

Another challenge is related to understanding the importance of family members. This is how Ariella, the mother of a soldier with a head injury describes it: *The injured person is wrapped up like cotton wool, but the family is also coping, and they are the engine for the rehabilitation of the wounded family member.* Mentors sometimes struggled to recognize that connecting with family members serves as a bridge to the injured person, and that maintaining such contact is a central part of their role rather than a form of “malpractice.” This issue was repeatedly discussed in the supervision groups and was especially evident in severe injuries, such as SCI and TBI, where family members often serve as primary caregivers.

#### 4.3 Findings of the linguistic analysis

The linguistic analysis added a further layer to the findings from the thematic analysis. Specifically, the linguistic analysis of interview transcripts (12,362 words were analyzed) identified 463 instances of linguistic intensification: 192 instances of the use of adverbial intensifiers and 271 instances of repetition. The ATLAS qualitative content analysis identified 71 instances of intensification in the utterances that addressed the caregivers’ portrayal of program. The most interesting finding is that analysis of the emotionally colored language via intensification showed that most of the identified utterances (82%) described the program’s contribution to the wounded within the *emotional* dimension; 13 utterances (18%) portrayed the program’s contribution to the wounded within the *functional* dimension. The emotional dimension was realized through the motifs of transformation in the injured person’s feelings from despair to hope, envisioning the mentor as a source of hope (60%), from alienation to rebuilding trust (28%), and from helplessness to a sense of self-efficacy (6%). For instance, one of the caregivers described how the mentor served as a source of hope: *“Suddenly he comes and tells you how things will look, it gives you hope, but also a sense of seeing the future, seeing what it looks like…and really, I think it gives my son lots and lots of strength to see him as such.”*

In addition, four instances (6%) of intensification were observed in the utterances portraying the contribution of the program to the wounded in the *functional* dimension. These utterances described fostering physical functional independence: *“and speech therapy and so on and so forth. He simply sat and helped him, so he did. He helped him a great deal.”* They also mentioned receiving all the necessary information regarding the realization of the wounded’s rights due to the mentor. To conclude, the linguistic analysis brought into relief the different domains of peer mentorship assistance–yet it mostly underscored the elements of functional, and emotional support.

## 5 Discussion

The present study examined the benefits and barriers of hospital-based peer-mentoring of severely injured veterans during the October 7th War from the perspective of the injured and caregivers. The study revealed that peer-mentoring facilitates recovery across three domains: functional, social–emotional, and cognitive. Specifically, on the functional domain, peer-mentorship facilitates independent physical functioning in the activities of daily living (ADL), exercising rights, and participation in the community. On the social–emotional level, peer-mentorship was found to promote a sense of hope, belonging, recognition and trust–which facilitates a sense of self-efficacy to face the challenges of rehabilitation alongside the re-entry to the public sphere. On the cognitive domain, peer-mentorship was found to foster a “competent mindset” by facilitating a state-of-mind of autonomous choice, effective communication with others, insight and normalization and attending the challenged self-identity.

The study echoes previous findings suggesting that community-based peer mentoring offers informational and emotional support (27). Rehabilitation during ongoing armed conflict is not unique to the Israeli context: the conflicts in Iraq and Afghanistan, and the continuing war in Ukraine, have produced comparable cohorts of severely injured service members undergoing rehabilitation while hostilities persist. What is relatively novel in the program examined here is its configuration: structured peer mentorship delivered within acute inpatient rehabilitation, by previously injured veterans formally re-recruited to serve as mentors in reserve duty, at a time of mass casualty influx. Precisely because this configuration responds to a problem shared across contemporary conflicts, the present findings, including the implementation barriers, are likely to be of practical use well beyond Israel. The novelty of the current study lies in its broader conceptualization that unfolds the ways in which functional, socio-emotional, and cognitive domains are intertwined in the facilitation of injured persons’ recovery. Figure 2 displays the study’s model, highlighting the rehabilitation process through peer mentoring. The study’s findings may be interpreted through two theoretical frameworks: Self-Determination Theory (SDT), which emphasizes the satisfaction of basic psychological needs as a basis for intrinsic motivation and effective functioning, and Social Learning Theory (31), which highlights the role of learning from a model.

**Figure 2:**
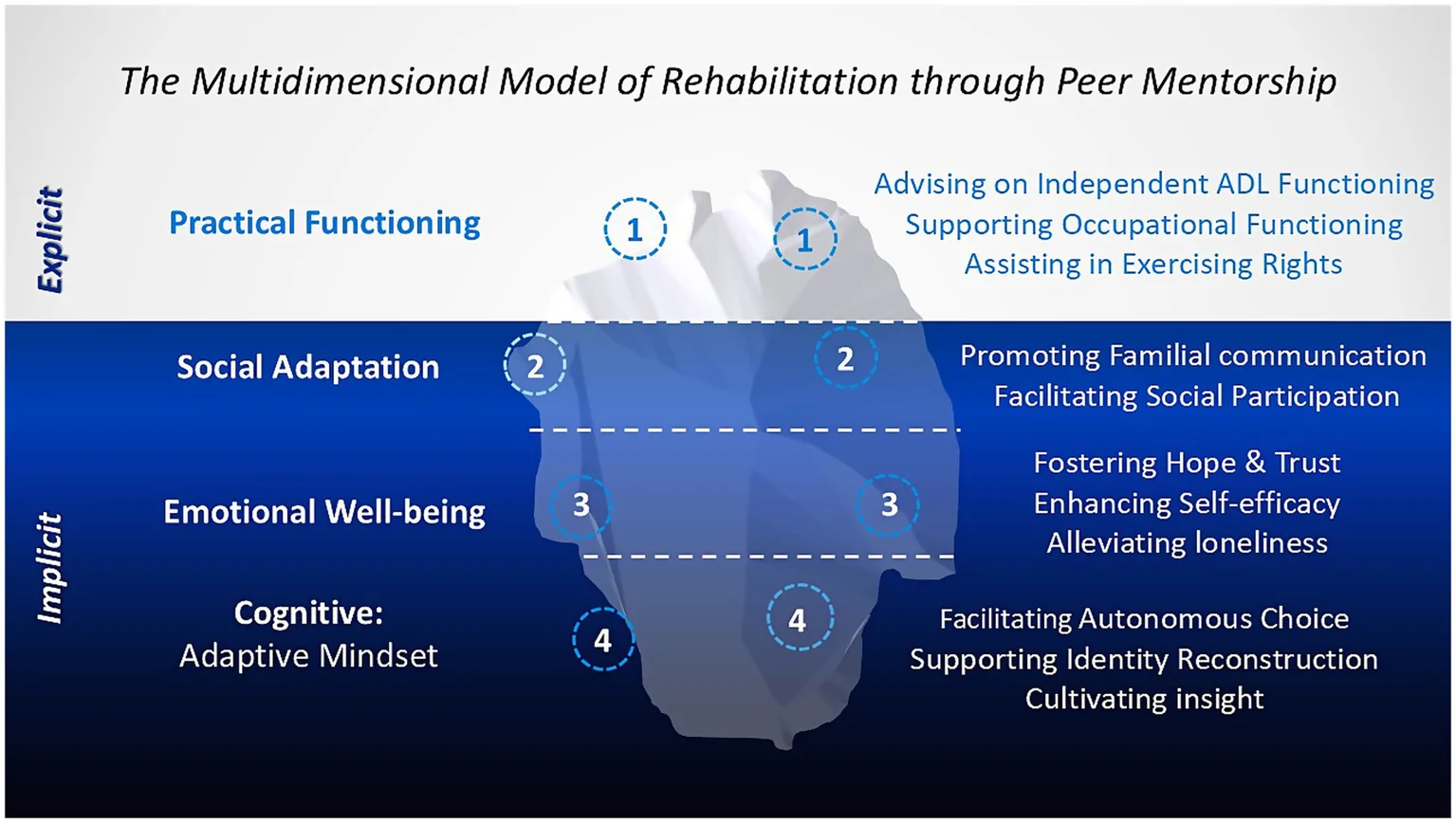
The Multidimensional Model of Rehabilitation through Peer Mentorship.

Figure 2 employs an iceberg metaphor to represent the multidimensional operation of peer mentorship. The waterline distinguishes the explicit dimension of the mentoring relationship from those dimensions that remain implicit. Situated above the waterline is the domain of practical functioning, the dimension participants articulated most readily and the one by which the program is ostensibly defined: advising on independent functioning in activities of daily living, supporting occupational functioning, and assisting in exercising rights. The remaining domains are situated below the waterline and are ordered by decreasing visibility. In the figure, the social–emotional domain reported in the Findings is represented as two distinct strata, as its social and emotional components differ in the degree to which they are explicitly articulated within the mentoring relationship. The upper stratum, social adaptation, encompasses the promotion of familial communication and the facilitation of social participation; the lower, emotional well-being, encompasses the fostering of hope and trust, the enhancement of self-efficacy, and the alleviation of loneliness. At the greatest depth lies the cognitive domain of an adaptive mindset, encompassing the facilitation of autonomous choice, support for identity reconstruction, and the cultivation of insight. The model thus proposes that the practical assistance visible above the waterline constitutes the point of entry to the less visible domains beneath it, in which much of the recovery process described by participants was located.

Supporting injured soldiers’ psychological needs through peer mentoring.

Self-Determination Theory (SDT) posits that optimal functioning depends on the extent to which the social environment supports three basic psychological needs: autonomy, namely a sense of ownership over one’s actions; competence, namely a sense of mastery; and relatedness, namely a sense of meaningful connection to others. When these needs are supported, motivation becomes more autonomous and internalized, which in turn promotes persistence, psychological well-being, and adaptive functioning (30). Indeed, recent evidence further suggests that these processes remain highly relevant in demanding contexts, including military settings (48).

From this perspective, peer mentorship may be understood as a need-supportive environment: it appeared to promote competence by offering concrete guidance, modeling effective coping and rehabilitation strategies, and strengthening injured soldiers’ sense of hope and self-efficacy; relatedness by providing emotionally credible connection grounded in shared lived experience; and autonomy by encouraging personal choice, providing insight, supporting identity reconstruction and ownership of the recovery process. As such, these processes suggest that peer mentorship did not merely provide practical assistance, but also supported the satisfaction of psychological needs, thereby fostering psychological well-being and adaptive functioning.

Fostering self-efficacy among injured soldiers: understanding the processes of peer mentoring through social learning theory.

Bandura’s Social Learning Theory (31) emphasizes the role of modeled learning in the development of self-efficacy, defined as individuals’ beliefs in their ability to achieve desired levels of performance. These beliefs shape thought, emotion, motivation, and behavior. According to the theory, people acquire knowledge, skills, and coping strategies by observing others, particularly models they perceive as similar to themselves. In line with the Model–Observer Similarity Hypothesis (31), greater perceived similarity in characteristics such as age, background, expertise, or gender increases both learning from the model and its effect on self-efficacy. Similarly (49), showed that learners are drawn to models who possess skills they aspire to acquire and learn not only from their actions but also from their ways of thinking. From this perspective, wounded IDF veterans who have undergone successful rehabilitation may serve as credible peer models for newly injured soldiers, enabling them to acquire new skills and strengthen self-efficacy while adapting to injury. This is especially important given evidence that self-efficacy is positively associated with mental health, with increased self-efficacy linked to lower psychological distress and more adaptive emotional functioning (50).

Bandura (31) identified four main sources of self-efficacy, all of which were reflected in the current findings. First, *mastery experiences* strengthen efficacy through personal success. Consistently (51), identified mastery experience as the strongest source of self-efficacy in health-related context. In the present study, peer mentors promoted such experiences by offering practical, experience-based guidance that helped injured soldiers achieve early successes in coping with functional challenges, particularly during hospitalization, when confidence is often fragile. Second, *vicarious experience* develops through observing similar others succeed. In line with Bandura’s argument, participants described similarity in injury profile as central to both learning from the mentor and strengthening self-efficacy, which supports the unit’s matching approach. Observation, however, extended beyond visible functioning to coping-related ways of thinking, including perspectives that emphasized agency rather than victimhood.

Third, *social persuasion* strengthens efficacy through encouragement from credible and knowledgeable others. Participants described the mentor’s encouragement as especially persuasive because it came from someone who had undergone a similar injury. Mentors were also described as helping injured soldiers set a realistic and gradual “effort bar” in rehabilitation, thereby supporting incremental progress. This aligns with Bandura’s claim that verbal persuasion is effective when paired with realistic conditions for success rather than empty reassurance. Family members similarly described mentors as helping shape realistic expectations. Finally, Bandura argued that efficacy beliefs are shaped by *physical and emotional states*. Consistent with this, participants described engagement in sport with the mentor as helping regulate physical arousal and strengthen self-efficacy, in line with evidence that positive affective states contribute to efficacy beliefs (51). Overall, the findings suggest that peer mentoring promotes self-efficacy through all four of Bandura’s pathways: mastery experience, vicarious learning, social persuasion, and physical and emotional regulation. Peer mentorship therefore appears to hold significant potential for supporting the recovery of newly injured soldiers. Yet its barriers should be carefully considered.

Attending the Barriers of Peer-mentoring in Healthcare Settings.

The study identified challenges in peer mentoring across three domains: technical-functional (hospital-related rehabilitation overload, adequacy of matching and timing), emotional (managing rejection, shattering the illusion of complete recovery, appearance-related avoidance), and cognitive (discrepancies in worldview and recognizing family’s role during the acute phase of hospitalization) - some of which have not been previously documented in the literature. To date, research on peer mentorship for veterans, conducted mostly in community-based settings, indicates that implementation is shaped by multiple interrelated challenges. These include role ambiguity, mentor-mentee mismatching, and insufficient supervision (15, 52). Implementation may also be hindered by practical barriers, including transportation, scheduling difficulties and maintaining participants engagement (53). Additionally, although shared military identity and lived experience appear to facilitate connection, the literature remains heterogeneous, making it difficult to establish definitive best practices for implementation of peer-mentoring, thus further study is necessary (54).

The present study extends the existing literature by examining peer mentorship within an acute hospital inpatient setting, thereby shedding light on implementation challenges that require particular attention in this context. While, in line with previous research, careful matching and the management of technical issues emerged as important, the findings also reveal several challenges that have received little attention to date. These include the emotional challenges faced by injured individuals and their families, including ambivalence in response to the mentor’s physical appearance and the conflict with a defensive mechanism that anticipated full recovery as well as the mentor’s emotional burden, especially in coping with rejection and the need to ‘reach out’. Interestingly, mentors assigned to several injured individuals appeared to cope better with rejection from one mentee, as they simultaneously experienced success with another one. In Bandura’s terms, such parallel mastery experiences may have strengthened mentors’ self-efficacy, thereby supporting their persistence in their duty. The findings further highlight challenges arising from differences in worldviews, emphasizing the need to underscore to mentors that individuals differ in their rehabilitation needs. Moreover, the study suggests that the type of injury is a key consideration in determining the timing of mentor induction, as severe or less visible injuries, such as TBI or SCI, may require a longer adjustment period and a more gradual process. Taken together, these findings suggest that peer-mentorship programs implemented within acute hospital settings require unique clinical considerations. These include ensuring an accurate mentor–mentee match, determining the appropriate timing for initiating mentorship, securing family consent while addressing the emotional difficulties involved in joining the program, and providing mentors with initial training as well as ongoing supervision and support. Such support is particularly important given that mentors may encounter rejection, which can cause emotional distress and may affect their well-being and professional functioning.

An additional key implication involves mentor identity and mentor–mentee matching. Although prior research highlights the importance of matching by injury profile, gender, and service background (22, 28), the present study suggests that the mentor’s personal and emotional maturity is equally critical. Even when age or socioeconomic differences existed, connection was established when the mentor demonstrated strong empathic capacity. This finding is consistent with prior research among military populations showing that attitudes toward the out-group may shift under conditions of close relationship (55). As such, mentor selection should assess not only injury type, but also the mentor’s personal readiness, including the extent to which they have emotionally processed their own injury. This interpretation aligns with research showing that perceived similarity promotes experience sharing and empathy, yet exposure to another person’s distress may reduce empathy unless one’s own similar experience has been sufficiently processed (56). Consistently, mentors who had processed their own injury were better able to convey empathy and build connection. This may explain why some mentors struggle to connect despite a similar injury match. Thus, beyond similarity in injury profile and background, the mentor’s readiness and empathic capacity appear to be the most important key factor in effective mentoring.

## 6 Limitations and future directions

This study has certain limitations. There is no gender equality, with most of the injured being men and most of the parent caregivers being women. However, this represents the current situation regarding enlistment in combat units. Follow-up studies should focus on the experiences of wounded female soldiers. Further research should also examine the contribution of peer-mentoring to the mentors themselves. Finally, since this study is qualitative in nature, examination of the research questions could be expanded by means of quantitative research.

The study was also cross-sectional, capturing perceptions at a single point between 6 months and 2 years post-injury and relying on retrospective accounts of the earliest phase of hospitalization. A longitudinal interview design, following mentees, caregivers and mentors across the rehabilitation trajectory, would clarify how perceptions of mentoring change over time and through the process of recovery. This appears particularly important for the barriers identified here, several of which are explicitly time-dependent: whether resistance to encountering a mentor with SCI recedes as the injury is internalized, and whether the practical value of mentoring gives way to relational or identity-related value as rehabilitation progresses, are questions that require further studies.

## 7 Importance and implications

The study indicates that peer mentoring significantly contributes to the recovery of the injured and their family across three domains of functioning: physical, social–emotional, and cognitive. The findings are relevant in the global context, which is unfortunately characterized by recurrent continuous wars. The importance of the study lies in the fact that in the context of mass casualties, such as in the October 7th War, there is an urgent need to expand the range of responses, and to base interventions on a broad ecological perspective (57). Implementing peer mentoring models will enable hospitals, mental health frameworks, and rehabilitation centers to offer additional interventions to provide holistic rehabilitation to all in need.

## Data Availability

The raw data supporting the conclusions of this article will be made available by the authors, without undue reservation.

## Author’s note

This paper integrates partners with lived experience as co-authors. *Lived experience* refers to the personal knowledge of individuals who have been directly affected by traumatic events, and these individuals are currently serving in roles within the Unit Headquarters. We value this form of knowledge not only as a source of insight into trauma care, but also as a means to foster more inclusive, ethical, and effective research and clinical practices.

## References

[1] George-Levi S Faverman L Galin-Lonchich Y Ben-Gal Dahan A. Beyond meaning: hope and secondary trauma in Israeli therapists after the October 7th massacre. Front Psychol. (2025) 16:1594885. doi: 10.3389/fpsyg.2025.1594885, 40535191 PMC12175434

[2] Katsoty D Greidinger M Neria Y Segev A Lurie I. A prediction model of PTSD in the Israeli population in the aftermath of October 7th, 2023, terrorist attack and the Israel–Hamas war. Isr J Health Policy Res. (2024) 13:63. doi: 10.1186/s13584-024-00644-6, 39472957 PMC11520871

[3] IDF Spokesperson IDF fallen and wounded in the wa. War journal (2026). Available online at: https://www.idf.il/ (Accessed April 24, 2026).

[4] Franco Z Ruffalo L Curry B Gollin-Graves M Ahamed SI Winstead O . Impact of veteran-led peer mentorship on posttraumatic stress disorder. J Trauma Stress. (2024) 37:617–30. doi: 10.1002/jts.23038, 38635149

[5] Elster EA Butler FK Rasmussen TE. Implications of combat casualty care for mass casualty events. JAMA. (2013) 310:475–6. doi: 10.1001/jama.2013.167481, 23925612

[6] Shiloh S Heruti I Leichtentritt R. A common-sense model of injury perceptions. J Health Psychol. (2016) 21:1516–26. doi: 10.1177/1359105314557876 25432457

[7] Fitzpatrick KF Pasquina PF. Overview of the rehabilitation of the combat casualty. Mil Med. (2010) 175:013–7. doi: 10.7205/milmed-d-10-00159, 23634473

[8] Capaldi VF Zembrzuska HD. Thrust into the breach: psychiatry in a combat zone within 1 year of residency completion. Acad Psychiatry. (2015) 39:410–5. doi: 10.1007/s40596-015-0283-3, 25636255

[9] Kim SY Soumoff AA Raiciulescu S Kemezis PA Spinks EA Brody DL . Association of traumatic brain injury severity and self-reported neuropsychiatric symptoms in wounded military service members. Neurotrauma Reports. (2023) 4:14–24. doi: 10.1089/neur.2022.0063, 36726873 PMC9886188

[10] Gilber O Spector-Marzel G Levy O Dekel R. Between fracture and change: the male identity of former soldiers suffering from post-traumatic stress following exposure to combat. Soc Welfare. (2018) 38:401–27.

[11] Schulz RN Jannace KC Cooper DB Luken ML Michel AR Sparling TL . Outpatient rehabilitation utilization after major limb loss in adults receiving care in the military health system from 2001 to 2017. Arch Phys Med Rehabil. (2025) 106:366–78. doi: 10.1016/j.apmr.2024.09.017, 39384117

[12] Sparling T Iyer L Pasquina P Petrus E. Cortical reorganization after limb loss: bridging the gap between basic science and clinical recovery. J Neurosci. (2024) 44:e1051232024. doi: 10.1523/jneurosci.1051-23.2023, 38171645 PMC10851691

[13] Woodruff SI Galarneau MR McCabe CT Sack DI Clouser MC. Health-related quality of life among US military personnel injured in combat: findings from the wounded warrior recovery project. Qual Life Res. (2018) 27:1393–402. doi: 10.1007/s11136-018-1806-7, 29450855

[14] Hawkins BL. Understanding contextual influences of community reintegration among injured servicemembers. J. Rehabil. Res. Dev. (2015) 52:527–42. doi: 10.1682/JRRD.2014.08.0196, 26436882

[15] Kayes N Mudge S Bright F Boland P Cummins C McPherson K. Peer support after traumatic brain injury: a protocol for a randomised controlled trial. BMJ Open. (2023) 13:e069167. doi: 10.1136/bmjopen-2022-069167 PMC9906261 36750279

[16] Greden JF Valenstein M Spinner J Blow A Gorman LA Dalack GW . Buddy-to-buddy, a citizen soldier peer support program to counteract stigma, PTSD, depression, and suicide. Ann N Y Acad Sci. (2010) 1208:90–7. doi: 10.1111/j.1749-6632.2010.05719.x, 20955330

[17] Adler Ben-Dor IA Kraus E Goldfarb Y Grayzman A Puschner B Moran GS. Perspectives and experiences of stakeholders on self-disclosure of peers in mental health services. Community Ment Health J. (2024) 60:1308–21. doi: 10.1007/s10597-024-01287-2, 38730076 PMC11408566

[18] Lam G Chung EYH. Mental health services in Hong Kong: development of community-based inclusive development practice faced with the cultural barriers. Ment Health Rev J. (2022) 27:426–36. doi: 10.1108/mhrj-11-2021-0087

[19] De Dios Perez B Morris RP Craven K Radford KA. Peer mentoring for people with acquired brain injury: a systematic review. Brain Inj. (2024) 38:316–29. doi: 10.1080/02699052.2024.2310779, 38318794

[20] Kelly D Malka M. It's not just me, it's us, together: the embodied of the wounded healer in the role of sex trade survival mentors - a critical mentoring perspective. Int J Environ Res Public Health. (2023) 20:4089. doi: 10.3390/ijerph20054089 PMC10001645 36901100

[21] Brown AD Ross N Sangraula M Laing A Kohrt BA. Transforming mental healthcare in higher education through scalable mental health interventions. Global Mental Health. (2023) 10:e33. doi: 10.1017/gmh.2023.29, 37854434 PMC10579693

[22] Clark C Barrett B Frei A Christy A. What makes a peer a peer? Psychiatr Rehabil J. (2016) 39:74–6. doi: 10.1037/prj0000147, 25984734

[23] Blayer Y Plivetsky N. “with you on your way”: integrating disabled IDF volunteers in the rehabilitation process of disabled IDF veterans. Meyda'os. (2022) 98:48–57.

[24] Kersten P Cummins K Kaiser N McPherson K. Making sense of recovery after traumatic brain injury through a peer mentoring intervention: a qualitative exploration. BMJ Open. (2018) 8:e020672. doi: 10.1136/bmjopen-2017-020672 PMC6252636 30309988

[25] Hagaman A Warren HL Miller R Henderson C. Peer support service activity prevalence by setting: a nine-state survey of peer workers. Front Public Health. (2025) 13:1533051. doi: 10.3389/fpubh.2025.1533051, 40109427 PMC11922078

[26] Horn K Flinn RE Hagaman AM Zajac K Hoffman LA Poulsen MN . Systematizing peer recovery support services for substance use disorder: a taxonomy for measuring recovery milestones. Front Public Health. (2025) 13:1529078. doi: 10.3389/fpubh.2025.1529078 PMC12104978 40421364

[27] Magasi S Papadimitriou C. Peer support interventions in physical medicine and rehabilitation: a framework to advance the field. Arch Phys Med Rehabil. (2022) 103:S222–9. doi: 10.1016/j.apmr.2020.09.400, 33440133

[28] Hundt NE Robinson A Arney J Stanley MA Cully JA. Veterans’ perspectives on benefits and drawbacks of peer support for posttraumatic stress disorder. Mil Med. (2015) 180:851–6. doi: 10.7205/MILMED-D-14-00536, 26226526

[29] Li T Zheng L. When culture meets child rights: Confucian ethics and legal challenges in mental health protection for minors in China. Front Public Health. (2025) 13:1691211. doi: 10.3389/fpubh.2025.1691211, 41189974 PMC12580322

[30] Ryan RM Deci EL. Intrinsic and extrinsic motivation from a self-determination theory perspective: definitions, theory, practices, and future directions. Contemp Educ Psychol. (2020) 61:101860. doi: 10.1016/j.cedpsych.2020.101860, 42574925

[31] Bandura A. "Self-efficacy". In: Ramachaudran VS, editor. Encyclopedia of human Behavior, vol. 4. London: Academic Press (1994). p. 71–81.

[32] Creswell JW Poth CN. Qualitative Inquiry and Research design: Choosing among five Approaches. Thousand Oaks: Sage Publications (2016).

[33] Chung EYH Sin KKF Chow DHK. Qualitative outcomes and impact of a robotic intervention on children with autism spectrum disorder: a multiple embedded case study. Br J Occup Ther. (2024) 87:574–82. doi: 10.1177/03080226241252272, 40343357 PMC11887874

[34] Kassa EE Maree W Guez J Frei-Landau R. Family dynamics after sibling loss in a migration context: the experiences of Ethiopian bereaved siblings. European Journal of Psychotraumatology. (2026) 17:268–322. doi: 10.1080/20008066.2026.2683322 PMC13348109 42411701

[35] Granero-Molina J Ruiz-Fernández MD Fernández-Medina IM Núñez-Nagy S Suazo Galdames IC. Qualitative research applied to public health: new topics and insight. Front Public Health. (2024) 12:1371938. doi: 10.3389/fpubh.2024.1371938 PMC10877045 38379673

[36] Frei-Landau R Lifshitz E Guez J. Losing a Kidney to Save a Life: Meaning Making of Bereaved Altruistic Kidney Donors Post-Donation. Journal of Loss and Trauma, (2025) 1–24. doi: 10.1080/15325024.2025.2477524

[37] Norris D Schwartz KD Eichler M Tam-Seto L Mahar A Smith-Evans K . A qualitative study of the capabilities of family members of veterans living with operational stress injuries. Fam Relat. (2023) 72:2869–85. doi: 10.1111/fare.12801

[38] Frei-Landau R Schonfeld DJ. Digital death education to promote pre-service teachers’ grief literacy regarding childhood bereavement: A qualitative case study. Death Studies, (2025) 1–17. doi: 10.1080/07481187.2025.2468175 39976561

[39] Frei-Landau R. Teachers as caregivers of grieving children in school in the post-COVID-19 era: using the self-determination theory to conceptualize teachers’ needs when supporting grieving children’s mental health. Frontiers in Pediatrics, (2024) 12:1320106. doi: 10.3389/fped.2024.1320106 PMC11169661 38873582

[40] Frei-Landau R Guez J Etty L. Altruistic kidney donation following the death of a loved one—a coincidence or a post-traumatic growth? Death Studies, (2026) 1–12. doi: 10.1080/07481187.2024.2432283 39579258

[41] Fusch PI Ness LR. Are we there yet? Data saturation in qualitative research. Qual Rep. (2015) 20:1408–16. doi: 10.46743/2160-3715/2015.2281

[42] Boutton-Laor Y Muchnik-Rozanov Y Frei-Landau R. “Why Are You happy if your dad died?”: The social experiences of parentally bereaved children in elementary and middle schools. Children. (2026) 13:155. doi: 10.3390/children13010155 PMC12840442 41597163

[43] Braun V Clarke V. Reflecting on reflexive thematic analysis. Qual Res Sport Exerc Health. (2019) 11:589–97. doi: 10.1080/2159676x.2019.1628806

[44] Schieble M Vetter A Meacham M. A discourse analytic approach to video analysis of teaching: aligning desired identities with practice. J Teach Educ. (2015) 66:245–60. doi: 10.1177/0022487115573264

[45] Muchnik-Rozanov Y Tsybulsky A. Towards understanding the language of student teachers’ reflections in the context of professional identity development. Reflective Practice. (2019) 20:520–32. doi: 10.1080/14623943.2019.1642188

[46] Halliday MAK Matthiessen CMIM. An Introduction to Functional Grammar. 3rd ed. London: Hodder Arnold (2004).

[47] Kornbluh M. Combatting challenges to establishing trustworthiness in qualitative research. Qual Res Psychol. (2015) 12:397–414. doi: 10.1080/14780887.2015.1021941

[48] Loverre M Chirico A Cinque L Palombi T Alivernini F Lucidi F . A systematic review of self-determination theory’s application in military and police organizations. J Police Crim Psychol. (2025) 40:685–707. doi: 10.1007/s11896-024-09718-2

[49] Hoogerheide V van Wermeskerken M van Nassau H van Gog T. Model-observer similarity and task-appropriateness in learning from video modeling examples: do model and student gender affect test performance, self-efficacy, and perceived competence? Comput Hum Behav. (2018) 89:457–64. doi: 10.1016/j.chb.2017.11.012

[50] Rohde J Marciniak MA Henninger M Homan S Ries A Paersch C . Effects of a digital self-efficacy training in stressed university students: a randomized controlled trial. PLoS One. (2024) 19:e0305103. doi: 10.1371/journal.pone.0305103 PMC11527301 39480821

[51] Egele VS Klopp E Stark R. An empirical ranking of the importance of the sources of self-efficacy for physical activity. Health Psychol Behav Med. (2025) 13:2567322. doi: 10.1080/21642850.2025.2567322, 41064471 PMC12502103

[52] Shepardson RL Johnson EM Possemato K Arigo D Funderburk JS. Perceived barriers and facilitators to implementation of peer support in veterans health administration primary care-mental health integration settings. Psychol Serv. (2019) 16:433–44. doi: 10.1037/ser0000242, 30407053 PMC7905501

[53] Matthias MS Kukla M McGuire AB Damush TM Gill N Bair MJ. Facilitators and barriers to participation in a peer support intervention for veterans with chronic pain. Clin J Pain. (2016) 32:534–40. doi: 10.1097/AJP.0000000000000297, 26340653 PMC4794408

[54] Mercier JM Hosseiny F Rodrigues S Friio A Brémault-Phillips S Shields DM . Peer support activities for veterans, serving members, and their families: results of a scoping review. Int J Environ Res Public Health. (2023) 20:3628. doi: 10.3390/ijerph20043628, 36834328 PMC9964749

[55] Ben-Shalom U. Soldiers' in-group and out-group peer perception: contact and ethnic identity. Mil Psychol. (2012) 24:473–87. doi: 10.1080/08995605.2012.716267

[56] Israelashvili J Sauter DA Fischer AH. Different faces of empathy: feelings of similarity disrupt recognition of negative emotions. J Exp Soc Psychol. (2020) 87:103912. doi: 10.1016/j.jesp.2019.103912, 32127724 PMC7001982

[57] Notman-Schwartz A Dekel R. Expanding the range of interventions in bereavement due to the black sabbath and the Iron swords war: towards an ecological concept – op-ed. Soc Secur Bull. (2024) 123:331–47.

